# Gestational gigantomastia with fatal outcome

**DOI:** 10.4322/acr.2020.213

**Published:** 2020-11-20

**Authors:** Natalia Rakislova, Lucilia Lovane, Fabiola Fernandes, Emília Gonçalves, Quique Bassat, Sibone Mocumbi, Jaume Ordi, Carla Carrilho

**Affiliations:** 1 Barcelona Institute for Global health (ISGlobal), Hospital Clínic- Universitat de Barcelona, Barcelona, Spain; 2 Universitat de Barcelona, Hospital Clinic, Department of Pathology, Barcelona, Spain; 3 Maputo Central Hospital, Department of Pathology, Maputo, Mozambique; 4 Eduardo Mondlane University, Faculty of Medicine, Department of Pathology, Maputo, Mozambique; 5 Eduardo Mondlane University, Faculty of Medicine, Department of Ginecology and Obstetrics, Maputo, Mozambique; 6 Maputo Central Hospital, Department of Ginecology and Obstetrics, Maputo, Mozambique; 7 Centro de Investigação em Saúde de Manhiça (CISM), Maputo, Mozambique; 8 Catalan Institution for Research and Advanced Studies (ICREA), Barcelona, Spain; 9 University of Barcelona, Hospital Sant Joan de Déu, Pediatrics Department, Pediatric Infectious Diseases Unit, Barcelona, Spain; 10 Consorcio de Investigación Biomédica en Red de Epidemiología y Salud Pública (CIBERESP), Madrid, Spain

**Keywords:** Gigantomastia, Breast Diseases, Pregnancy, Pregnancy Complications, HIV

## Abstract

Gigantomastia is a rare disease defined by an extreme and rapid enlargement of the breast, generally bilateral. The majority of cases are reported in pregnant women. Ninety-eight cases of gestational gigantomastia have been identified in electronic databases, and those with fatal outcomes comprised only 2 cases (2%). Despite its benign nature, it can lead to severe complications and even death. Its etiology has not been fully elucidated, but it has been speculated that a hormonal component may play a role in the pathogenesis. Currently, treatment options are limited, and surgery is gaining importance, but it is often not feasible in low-resource settings. Herein, we describe a case of a 30-year-old HIV-positive female with no relevant past medical history, who died due to the complications of gestational gigantomastia at the Maputo Central Hospital, in Mozambique.

## INTRODUCTION

Gestational gigantomastia (GG), also known as gravidic macromastia, is a rare condition characterized by an exaggerated, rapid, and frequently disabling enlargement of the breasts. Less than 100 cases have been reported in the literature.^1^ Most frequently, GG has its onset in the first trimester of gestation.^1^ The disorder is particularly difficult to manage in low-resource settings where treatment options are limited, and breastfeeding remains important.^2^ In Mozambique, no case of GG has been previously reported.

The disease is usually bilateral. Although there is no universally accepted definition of GG, it is generally defined as an increase in breast weight that exceeds 3% of the total body weight.^3^ Another proposed definition includes the requirement of 800 to 2000 grams of tissue removed at surgery from each breast.^4^ ^,^ ^5^ While GG etiology is still unclear, hypersensitivity to certain hormones (e.g. estrogen, progesterone, and prolactin) might play a role in its pathogenesis.^1^ This condition usually has a favorable prognosis after either medical or surgical treatment. Herein, we describe an exceptional case of GG with a fatal outcome in an HIV-infected patient and review the characteristics, diagnosis, and treatment of this rare condition.

## CASE REPORT

**Figure 1:**
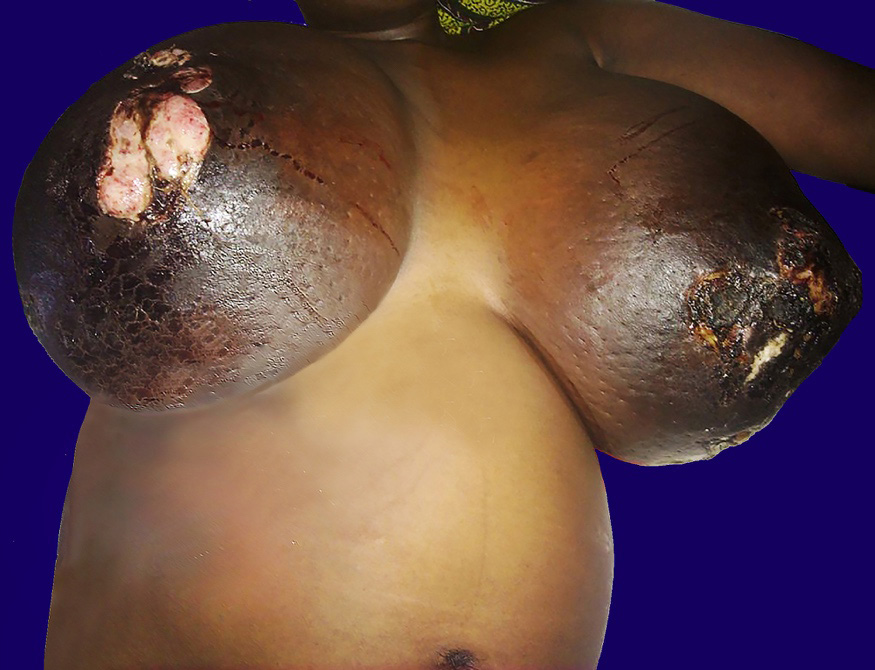
Gross view of the patient at 24 weeks of gestation in supine position showing a massive enlargement of both breasts with ulcers and necrosis of the overlying skin.

A 30-year-old woman, gravida 2, para 1, was admitted to the hospital at 24 weeks of gestation with a clinical diagnosis of inflammatory breast carcinoma performed at the emergency room. She was known to be HIV-positive and had been under antiretroviral treatment. However, the exact data of the diagnosis, CD4 counts, viral load, and the ART treatment duration was unknown. At 8-10 weeks of gestational age, the patient noticed a progressive enlargement of both breasts, which was initially painless, and then evolved with burning pain and marked discomfort. No regular prenatal visits were conducted during pregnancy. On physical examination, the patient was pale and afebrile; her pulse rate was 86 bpm, blood pressure was 100/74 mm Hg. Heart sounds were normal, and the chest was clinically clear. A noticeable enlargement and swelling of both breasts were perceived. The overlying skin showed extensive areas of necrosis and ulceration (Figure 1), with hyperesthesia.

There was no abnormal breast discharge or suppuration, and no axillary lymph nodes were palpable. The patient had normal temperature. Laboratory tests revealed anemia (hemoglobin was 6.0 g/dL (reference range [RR]; 11.9-14.3 g/dL). The leucocyte and platelet counts were normal, 7.96 x 10^3^/µL (RR 4.5-10.5 x 10^3^/µL) and 303 x 10^3^/µL (RR 202-324 x 10^3^/µL), respectively. The HIV viral load, CD4 counts, inflammatory markers, as well as the endocrine workup, including serum prolactin levels and autoantibody testing for autoimmune diseases, could not be performed. The obstetric ultrasound scan showed two fetuses with normal heart activity, and no evident malformations. Upon the clinical findings, a diagnosis of bilateral GG was established. Breast reduction was not recommended due to the potential surgical complications, and the patient underwent conservative treatment with antibiotics (penicillin G) for 10 days. In spite of the medical treatment, the ulcers progressively increased in size and caused heavy bleeding. The patient lost between three and four liters of blood from the breasts' ulcers, and died on day 13 of hospitalization, despite receiving several blood transfusions. From a clinical point of view, the most likely immediate cause of death was hypovolemic shock.

## AUTOPSY FINDINGS

**Figure 2:**
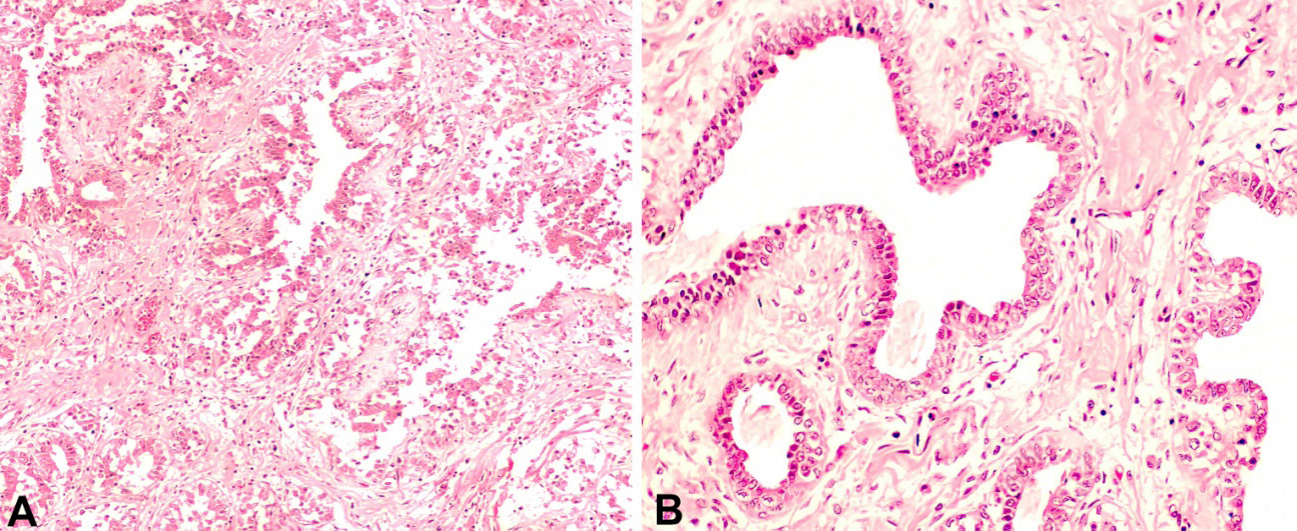
Photomicrograph of the breast parenchyma shows stromal edema, and ductal hyperplasia with mild dilation of breast ducts, with no signs of malignant transformation. **A** – Intralobular ducts are abundant and tortuous (H&E, 100X); **B** – Mild dilation of breast ducts. Scant inflammatory cells are seen in the stroma (H&E, 200X).

A complete autopsy was requested as a routine procedure for all maternal deaths at Maputo Central Hospital (MCH) and performed after the informed consent was obtained. The breasts' overlying skin showed extensive ulceration with remains of blood; no suppuration was noted. Histological changes in the skin of the breast included thrombi in the superficial dermal vessels and extensive ulceration of the epidermis. The most profound section of the breast parenchyma showed stromal edema, extensive ductal hyperplasia with mild dilation of ductal structures, and the absence of significant inflammatory infiltrate (Figure 2). No malignant transformation was identified.

The remaining internal organs were pale. Both kidneys showed reverse corticomedullary differentiation (“shock kidneys”). The spleen capsule was diffusely wrinkled, but the parenchymal consistency was normal. On histological evaluation, neither significant inflammatory infiltrate nor other signs of sepsis were recognized in the spleen or any other of the examined organs/tissues.

The uterus contained a twin pregnancy with a monochorionic diamniotic placenta normally inserted, clear amniotic fluid with normal membranes, and two fetuses, one female and one male, with no evidence of malformations. No macroscopic or microscopic abnormalities were observed in the placenta. The autopsy final diagnoses included: 1) hypovolemic shock as the direct cause, and; 2) bleeding cutaneous breasts' ulcers as the intermediate cause, and; 3) GG as an underlying cause of death.

## DISCUSSION

We present in this report an extremely unusual case of a 30-year-old, HIV-positive patient who experienced a progressive, massive enlargement of both breasts during the first trimester of her second pregnancy, which eventually caused extensive ulceration of the overlying skin with severe hemorrhage and death due to hypovolemic shock. GG is an exceptionally rare condition that complicates between 1 per 28,000 and 1 per 100,000 pregnancies.^6^ The disease usually presents in the first trimester of gestation, as in the present case.^7^ ^,^ ^8^ The clinical history is consistent with other severe reported cases.^6^ ^,^ ^9^ Patients with GG may develop breast ulceration, hemorrhage, cellulitis, pain, decreased mobility, and can be also be psychologically affected.^6^ ^,^ ^10^ ^-^ ^12^ The etiology remains unclear; however, it is believed to arise as a result of over-sensitivity or overproduction of pregnancy-related hormones, such as prolactin.^7^ ^,^ ^13^ Alternatively, autoimmunity may also play a role in its pathogenesis;^14^ indeed, the simultaneous presence of autoimmune diseases has been reported in several cases.^15^ ^,^ ^16^ Nevertheless, in the majority of reports, laboratory workup rarely demonstrates any abnormality.^17^

The histological analysis of breast tissue often reveals pregnancy-lactation changes such as glandular hyperplasia without atypia, overgrowth of connective tissue, and tissue fibrosis, with the absence of prominent inflammatory infiltrate, as in our case.^18^ Although the condition is completely benign, the clinical presentation may mimic malignancy, as was initially suspected in our case. Occasionally, malignant tumors can cause massive enlargement of the breasts mimicking gigantomastia (two cases of invasive ductal carcinoma, two cases of lobular carcinoma in situ,^19^ and occasional lymphomas,^20^ but most of these cases are not related to pregnancy). The differential diagnosis of GG may also include the phyllodes tumor and giant fibroadenoma, usually unilateral and not associated with pregnancy either.

The treatment depends on the severity of the signs and is usually initiated with bromocriptine. However, most patients do not respond and require surgery (reduction or mastectomy).^21^ Remarkably, once GG has occurred, recurrences are likely to occur in subsequent pregnancies unless surgery is performed.^18^ ^,^ ^22^ However, the management of GG is difficult in low-resource settings where many pregnant women have limited access to essential health services. In addition, the disease has more serious consequences because breastfeeding is of utmost importance in these settings.

The majority of GG reported severe cases with a similar clinical course had a favorable outcome after surgery.^6^ The main complications in the reported cases of severe GG are associated with skin ulceration, bleeding and necrosis.^6^ ^,^ ^9^ ^-^ ^12^ ^,^ ^23^ ^-^ ^25^ Indeed, breast ulceration, infection, and bleeding are potentially fatal complications and constitute absolute indications for surgical treatment. Unfortunately, urgent surgical intervention was not feasible in our case due to logistic factors, and the patient died due to hemorrhage.

This maternal death was preventable. On the one hand, regular prenatal consultations would have allowed the early management of GG. However, many Mozambican women do not have access to prenatal care,^26^ and those with access often delay prenatal clinic consultations, hampering the prevention and treatment of preventable pregnancy complications.^27^ On the other hand, this patient probably would have survived if logistical conditions allowed urgent high-quality surgical intervention. In this regard, more resources are needed to support public hospital infrastructure and improve the quality of care provided to pregnant women.^28^

We conducted an electronic search of PubMed and Scopus databases for all original articles, case reports, and case series on GG from 1951 to 2020. The search strategy included the keywords “gigantomastia” or “macromastia”, in combination with “gestational” or “pregnancy”. The articles with abstract and/or full text published in other languages were translated into English. From 314 initial results, we removed all the duplicates, misclassified review articles, and articles without abstract or full text available for revision. As a result, we obtained 109 studies. After a systematic revision of abstract and/or full text, we further excluded 25 studies. Of them, 20 reports focused on gigantomastia due to other causes (idiopathic, autoimmune, or puberty-related), whereas five were unrelated to GG (breast reconstruction surgery after malignancy). Thus, we were left with 84 studies that altogether reported 98 cases of GG.

Out of 98 reported GG cases, only two cases of maternal deaths directly related to GG have been identified.^9^ ^,^ ^29^ Therefore, the maternal mortality rates due to GG are extremely low; 2% (2/98). Table 1 shows the summary of clinical, laboratory, and treatment characteristics in previously described GG cases with fatal outcome. One of the patients died in 1984 shortly after a surgical abortion and had a concomitant debilitating disease (immunoendocrine insufficiency syndrome).^29^ The most recently reported case of GG with fatal outcome occurred in Nigeria in 2013.^9^ The woman died as a consequence of severe sepsis originated from the infected breast skin ulcer that progressed into renal failure and multi-organ dysfunction syndrome. GG is a progressive disease; hence, a good outcome depends on prompt diagnosis and treatment.

**Table 1:** Summary of gestational gigantomastia (GG) cases with fatal outcome

Reference	Age	Clinical presentation	Laboratory findings	Histology/microbiology	Treatment and outcome
Ibrahim et al.^9^	18	Pregnant woman with immunoendocrine insufficiency syndrome, admitted with enlargement of the mammary glands.	High blood prolactin level	Breast biopsy: proliferation of intralobular connective tissue and ducts; marked interstitial edema	The patient developed severe adrenal insufficiency due to the use of parlodel Surgical abortion was conducted. Death occurred hours after the surgery.
Zaĭrat’iants^29^	27	Admitted at 20 weeks of gestation with bilateral enlargement of the mammary glands, massive ulcerations and necrosis of the lower pole of the breasts. Discrete axillary lymphadenopathy was present.	Hemoglobin of 8 g/dL Neutrophilia, elevated prolactin and luteinizing hormones Blood culture: repeatedly positive for Staphylococcus aureus	Breast biopsy: chronic non-specific inflammation Breast culture: *Proteus spp*	Surgery was declined by the patient. At 22 weeks of gestation patientdelivered a dead fetus, developing severe sepsis within the next 48 hours. The patient died of sepsis

The relation between GG and HIV-associated immunosuppression is not clear. To our knowledge, no case of GG has been reported in an HIV-positive woman. Only a single case of non-gestational gigantomastia in an HIV patient has been reported previously in a nulliparous woman from India.^30^ This patient was not pregnant and had gigantomastia associated with bilateral axillary accessory breast and right breast fibroadenoma. As many as 70% of HIV-infected people worldwide live in sub-Saharan Africa,^31^ it is reasonable to assume that the coexistence of the two conditions is casual.

## CONCLUSION

GG is a rare condition. The majority of women with GG has a favorable outcome. However, its early diagnosis is of extreme importance since its complications may cause maternal and subsequent fetal death. Management of the most severe forms of GG is particularly problematic in low-resource settings. The relationship between HIV and GG is likely to be casual.
